# Investigating the Role of the Primary Motor Cortex in Musical Creativity: A Transcranial Direct Current Stimulation Study

**DOI:** 10.3389/fpsyg.2018.01758

**Published:** 2018-10-01

**Authors:** Aydin Anic, Kirk N. Olsen, William Forde Thompson

**Affiliations:** ^1^Department of Psychology, Macquarie University, Sydney, NSW, Australia; ^2^Centre for Elite Performance, Expertise and Training, Macquarie University, Sydney, NSW, Australia; ^3^Australian Research Council Centre of Excellence in Cognition and its Disorders, Macquarie University, Sydney, NSW, Australia

**Keywords:** creativity, expertise, musical improvisation, primary motor cortex, transcranial direct current stimulation

## Abstract

Neuroscientific research has revealed interconnected brain networks implicated in musical creativity, such as the executive control network, the default mode network, and premotor cortices. The present study employed brain stimulation to evaluate the role of the primary motor cortex (M1) in creative and technically fluent jazz piano improvisations. We implemented transcranial direct current stimulation (tDCS) to alter the neural activation patterns of the left hemispheric M1 whilst pianists performed improvisations with their right hand. Two groups of expert jazz pianists (*n* = 8 per group) performed five improvisations in each of two blocks. In Block 1, they improvised in the absence of brain stimulation. In Block 2, one group received inhibitory tDCS and the second group received excitatory tDCS while performing five new improvisations. Three independent expert-musicians judged the 160 performances on creativity and technical fluency using a 10-point Likert scale. As the M1 is involved in the acquisition and consolidation of motor skills and the control of hand orientation and velocity, we predicted that excitatory tDCS would increase the quality of improvisations relative to inhibitory tDCS. Indeed, improvisations under conditions of excitatory tDCS were rated as significantly more creative than those under conditions of inhibitory tDCS. A music analysis indicated that excitatory tDCS elicited improvisations with greater pitch range and number/variety of notes. Ratings of technical fluency did not differ significantly between tDCS groups. We discuss plausible mechanisms by which the M1 region contributes to musical creativity.

## Introduction

The ability of humans to generate novel ideas has fascinated scientists and philosophers for centuries. Such ideas are defined as *creative* when they involve both novelty and congruency (Benedek et al., 2014; Schwab et al., 2014). Novelty pertains to the originality of a specific idea; congruency is said to have occurred if an idea is contextually appropriate (Dietrich, 2004; Jauk et al., 2015). Other theorists include a third defining feature, arguing that acts can only be considered creative if they are also *non-obvious* (Boden, 2004).

Creative thought and behavior have significant implications for human life, and a large body of research has focused on understanding psychological mechanisms that underpin the creative process (e.g., Batey and Furnham, 2006; Simonton, 2010; Jauk et al., 2014; Tan et al., 2016). Over the past 10 years, researchers have begun to reveal the neural underpinnings of creative thought and action, employing methods such as fMRI (e.g., Limb and Braun, 2008) and EEG (e.g., Fink and Benedek, 2014). The present investigation used a novel method of online bihemispheric tDCS to investigate the neuroscience of creativity in the context of artistic enactment (Lucchiari et al., 2018). Specifically, tDCS was used to investigate the role of the M1 in creative piano improvisations performed by expert jazz pianists.

Musical improvisation represents an ecologically valid domain in which to explore the process of creativity because it requires novelty and continuous production of non-obvious but contextually appropriate passages of music (Bengtsson et al., 2007). Musical improvisation is a form of creative expression that can be defined as the composition or invention of music in real-time (Biasutti, 2015, 2017). Its implementation in real time means that no corrections can be made to creative output. Instead, improvisation is a temporally dynamic behavior that unfolds over time (Biasutti, 2015; Adhikari et al., 2016).

Improvisation plays a role in many genres of music but is most prominent in jazz, where musicians routinely generate novel melodies while observing complex rhythmic and harmonic templates that can be modulated to generate creative output (Biasutti and Frezza, 2009). In the context of neuroscientific research, musical improvisation is commonly used in studies designed to highlight brain networks involved in movement-based creativity (e.g., Bengtsson et al., 2007; Limb and Braun, 2008; Pinho et al., 2014). However, this research has focused primarily on regions of the brain involved in higher-order cognitive processing, without consideration of the M1. The M1 is usually known for low-level functions such as motor learning and consolidation of motor skills (Karok and Witney, 2013; Sosnik et al., 2014), yet its role in creativity is unknown.

Previous fMRI studies investigating the neural mechanisms that underpin musical creativity often report activation of the ECN (Bengtsson et al., 2007). The ECN is located in the frontal lobe and comprises the DLPFC, ACC, and AIC (Kuhn et al., 2013). The ECN mediates three distinct cognitive mechanisms associated with creativity: inhibition, working memory, and cognitive flexibility (Diamond, 2013; Sowden et al., 2015; Bendetowicz et al., 2017; Kenett et al., 2018). The DLPFC is particularly important in mediating attention, working memory, and goal-orientation (Boccia et al., 2015). The DMN is another neural network that underpins creative cognition in a musical context, yet operates in direct contrast to the ECN (Limb and Braun, 2008). The DMN is a combination of brain areas that include the vMPFC, the PCC, and the medial and lateral temporal lobes (Kuhn et al., 2013; Zhu et al., 2017). The vMPFC is of particular importance since it mediates mind wandering, future imagination, and is activated during tasks requiring musical creativity (Limb and Braun, 2008; Bashwiner et al., 2016; Kenett et al., 2018).

The PFC and specifically the DMN and ECN are of paramount importance to processes involved in creative cognition and behavior. This is true irrespective of the domain (e.g., artistic creativity vs. insightful problem solving; Gonen-Yaacovi et al., 2013). Moreover, the output of information processed by the DLPFC that forms part of the ECN branches to the motor cortices (Dietrich, 2004). To date, it is known that premotor cortices such as the pre-SMA and the ventral and dorsal counterparts of the premotor cortex (vPMC and dPMC, respectively) are involved in high-level motor planning and execution (Berkowitz and Ansari, 2008; de Manzano and Ullén, 2012; Sosnik et al., 2014). The pre-SMA is important in the temporal components of motor performance, whereas the vPMC and dPMC are both involved in selection and performance of novel motor outputs – features that are vitally important for creative improvisation in music performance (Chouinard and Paus, 2006; Hoshi and Tanji, 2007; Berkowitz and Ansari, 2008; de Manzano and Ullén, 2012).

It is clear from this brief review that some of the brain networks that underpin creative musical improvisations are associated with higher-order cognitive processing and motor planning. It is not yet clear, however, whether brain regions involved in low-level processes such as the M1 also play a significant role in creative musical performance. The M1 is important for motor acquisition, consolidation, and importantly for pianists, the orientation, velocity, and direction of movement in the arms and hands (Karok and Witney, 2013; Sosnik et al., 2014). Stimulation of the M1 also results in greater muscular synergies in the hand that enhance the ability to “generate novel patterns of muscular activity” (Waters-Metenier et al., 2014, p. 1037). Indeed, creativity in performances that require rapid changes in the muscular activity in the hand may be modulated by the M1 in two important ways. First, precise temporal and spatial hand movements are required for technically fluent piano performances. It is likely that with high levels of technical fluency comes the increased probability of realizing creative cognition through performed improvisation. Second, the M1 may function directly to control the implementation of motor plans arising from higher-order processes, acting as a neural gateway that impacts upon creative artistic enactment (Lucchiari et al., 2018). The present study was specifically designed to address these overarching hypotheses by investigating the role of the M1 in creative and technically fluent piano improvisations. The improvisations were performed by expert jazz pianists and creativity and technical fluency were adjudicated by expert musician adjudicators (see Anic et al., 2017 for pilot data).

The M1 is located in both hemispheres of the brain. The left hemispheric M1 tends to exert superior control of the right hand, whereas the right hemispheric M1 tends to exert superior control of the left hand (Brinkman and Kuypers, 1973; Vines et al., 2008b). The two hemispheres of the M1 are linked by an IHIC. When the left hemispheric M1 is activated during movement in the right hand, the right hemispheric M1 is naturally inhibited through the IHIC to facilitate right-handed movement (see also van den Berg et al., 2011).

In the present study, we investigated whether excitatory tDCS over the left hemispheric M1 enhances creativity and technical fluency of right-handed piano improvisations, when compared with inhibitory tDCS. If creativity is modulated by the M1, then creativity and technical fluency in right-handed piano improvisations should vary as a function of the type of tDCS administered to the left hemispheric M1. Specifically, we hypothesized that excitatory tDCS over the left M1 will result in an increase in creativity and technical fluency compared to inhibitory tDCS. A subsidiary aim was to examine the correlation between ratings of creativity and technical fluency by expert musician adjudicators.

## Materials and Methods

### Participants

Sixteen proficient jazz pianists (*M* = 24.1 years, *SD* = 7.2, 7 females) and three independent expert musical adjudicators were recruited for the study. Each musician produced 10 improvisations which were judged on two separate scales by all three adjudicators, resulting in a total of 960 ratings that were then subjected for analysis. Three of the 16 proficient jazz pianists reported to be left-handed; one reported to be mixed-handed. All pianists had undergone considerable formal musical training on piano (*M* = 9.6 years, *SD* = 4.4). A TMS safety screener with a series of health-related questions (e.g., do you, or anyone in your family, have epilepsy?) was administered to participants prior to tDCS stimulation to ensure the safe application of brain stimulation. All participants satisfied the requirements of the safety screener and no participant subsequently experienced adverse effects from the procedure. The pianists were reimbursed $50 or course credit for an undergraduate psychology unit for their participation. Three expert musicians were recruited as judges to rate the improvisations. All three judges had completed doctoral level education in music-related fields, had received an average of 12.67 years of formal music training (8, 10, and 20 years), and were experienced as adjudicators of music performances. The judges were independent in that they did not know each other and did not adjudicate the performances together. They were reimbursed up to $150 for the approximate time of 3 h to adjudicate the performances. All participants and judges gave informed consent and the study was approved by the Macquarie University Human Research Ethics Committee (HREC Reference number: 5201600392).

### Stimuli

**FIGURE 1:**
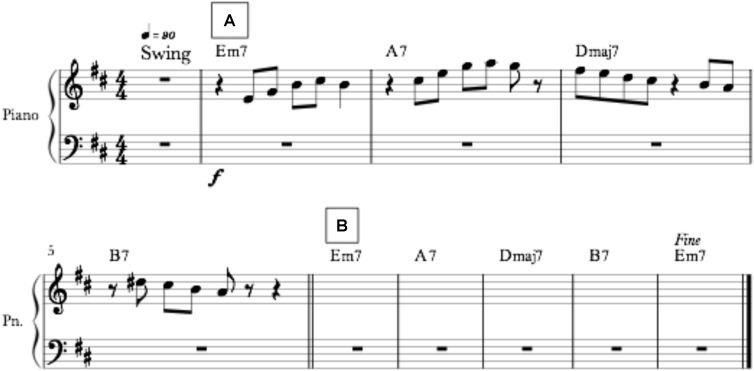
This figure shows an example of a musical score presented to participants in a trial. This example is written in the key of D major at a tempo of 90 beats per minute. The score shown at Section A marks the beginning of the sight-reading stage, where participants were instructed to play the displayed melody as accurately as possible. The score shown at Section B marks the beginning of the improvisation stage, where participants were instructed to play an improvisation based on the melody in the previous section and the harmonic structure of the music. Participants were instructed to play with their right-hand only.

Ten short pieces of music were custom-written by the first author (AA) for this study using *Notion* (Version 2.0.183) music software. These pieces were written to conform to a quintessential contemporary jazz style and provided participants with a musical context from which to perform their improvisations. All pieces incorporated an electronic drum kit, electric piano, grand piano from the *GarageBand* (Version 10.2.0) music software, and a live electric bass was played and recorded by the first author. Each musical piece contained 10 bars and lasted 30 s in total. An example score is shown in **Figure 1**. In each score, the first bar provided a four-beat count-in with an electronic high-hat cymbal on the drum kit to prepare participants for the beginning of the performance. Bars 2–5, labeled by the rehearsal marker “A” in **Figure 1**, contained a custom-written novel melody with the electronic drum kit, electronic piano and live electric bass acting as accompaniment for the harmonic and rhythmic qualities. In this “sight-reading” section, participants were instructed to reproduce the melody as accurately as possible, only on the treble clef and only with their right hand. Bars 6–10, labeled with the rehearsal marker “B” in **Figure 1**, comprised the improvisation section of the piece. In the section B – the “improvisation” section – the custom-written melody in section “A” was removed but the instrumental accompaniment remained to ensure rhythmic and harmonic quality and consistency. The participants were instructed to only use their right hand for both the sight-reading and improvisation sections. Seven of the 10 pieces were written in major key signatures (A, B, C × 2, D × 2, E*^b^*); the remaining three pieces were written in minor key signatures (B, D, G). All pieces were written in a 4:4 time signature with a swing feel at 90 beats per minute. See **Supplementary Material** for the scores of all 10 pieces.

### Equipment

The tDCS montage used in the present study comprised two saline-soaked electrodes diametric in charge: anode (positive) and cathode (negative) (Nitsche et al., 2003; Colombo et al., 2015). The anode charge heightens neural activity, whereas the cathode charge inhibits neural activity (Nitsche et al., 2003). An online bihemispheric tDCS configuration was implemented where both electrodes were placed on the scalp to stimulate the left and right M1 while each participant was engaged in the experimental task (see the “Experimental Design” subsection below for more detail). A study was conducted by Karok and Witney (2013) to determine the optimal tDCS configuration (placement of electrodes) and mode of tDCS (offline vs. online), and found that online bihemispheric tDCS is the optimal method for experiments designed to elicit significant changes in neural activity and subsequent behavior (see also Vines et al., 2008a; Waters-Metenier et al., 2014).

The online bihemispheric tDCS montage was set at 1.4 mA using two 25 cm^2^ electrodes to ensure a current density of 0.056/cm^2^, as recommended in Bikson et al. (2009). The saline-soaked electrodes (the anode and cathode) were attached onto an electroencephalogram (EEG) cap and worn by the participants with the tDCS device attached to the back of the cap. In accordance to the 10–20 EEG system, the electrodes were placed on the C3 and C4 electrode sites with the Cz electrode site situated on top of the scalp (Karok and Witney, 2013).

A 27-inch iMac was used to present each score to participants during each trial. The iMac was connected via a thunderbolt cable to a MacBook Air that played each piece of music and recorded each performance. All performances were conducted on a MIDI keyboard that was connected via USB to the MacBook Air. An additional MacBook Pro was used to run the Neuro-electrics Instrument Controller (NIC) (Version 1.4.10) that controlled the configuration and stimulation for the tDCS device. Once configured on the NIC software, the tDCS device was connected remotely to the MacBook Pro via Bluetooth and was attached to the cap on the back of the participants’ head. Each piece of music was played to participants through two external computer speakers.

### Experimental Design

Two tDCS stimulation conditions were developed for the experiment: Anodal-Left M1/Cathodal-Right M1 (excitatory tDCS group, *n* = 8) and Cathodal-Left M1/Anodal-Right M1 (inhibitory tDCS group, *n* = 8). These tDCS conditions were developed to target the right hand of participants. The 16 participants were pseudo-randomized into the two conditions to ensure an equal distribution of participants in the conditions. The 10 musical pieces were used to create 10 experiment trials (one piece per trial) that were further subdivided into two blocks. Block 1 contained five pieces (five trials or “takes”) to perform *without* tDCS stimulation. This served to evaluate a baseline rating of creativity and technical fluency under normal (no brain stimulation) performance conditions. Block 2 contained the remaining five pieces to perform during tDCS stimulation. The set of 10 pieces were initially randomly placed into the two blocks and to mitigate order effects, were further randomized within each block for each participant. In Block 2 – the stimulation block – either excitatory or inhibitory tDCS was applied to the participant’s left hemispheric M1, depending on the tDCS group they were placed in prior to the commencement of the experiment. Participants were blind to the type of tDCS stimulation they received and were tested individually in separate sessions. The duration of the experiment lasted approximately 90 min.

### Procedure

First, the TMS screener was administered to ensure that tDCS brain stimulation was safe to administer. Participants then gave informed consent to participate in the experiment and completed a demographic questionnaire. After this, participants completed five trials in the no-stimulation Block 1. Each trial consisted of two stages: familiarization and performance. The familiarization stage involved two practice runs for each piece of music in each trial. The first practice run involved the participant listening to the piece and following the melody in section “A” on the score *without* playing the piano. The melody in section A was played by a grand piano in the recording in addition to the musical accompaniment outlined above. The second practice run required the participant to play the displayed melody in section “A” with their right hand. The purpose of the familiarization stage was to ensure that participants were familiar with the piece of music in each trial.

After the familiarization stage of each trial, the performance stage commenced. The performance stage involved two complete attempts at each trial. The first performances in each trial in this stage were sent to the expert judges for adjudication, except for one trial from one participant who made significant errors in their improvisation and stopped playing. The purpose of allowing the participant to complete a second attempt at each trial was to reduce performance anxiety. The grand piano that played the melody in section “A” during the familiarization stage was removed in the performance stage. Each participant was instructed to play the melody in section “A” as accurately as possible. This enabled us to evaluate indicators of sight-reading accuracy such as timing (asynchrony of each note played relative to expected timing as stipulated in the score) and pitch-note accuracy (whether a correct note was played relative to each note in the score). They were instructed to perform their right-handed improvisations in section “B.”

After completing five trials in the no-stimulation Block 1, participants were administered the online bihemispheric tDCS montage specific to their allocated condition (Anodal-Left M1/Cathodal-Right M1 or Cathodal-Left M1/Anodal-Right M1). The first 30 s of stimulation involved a “ramp-up” period. All participants were stimulated for two and a half minutes (including ramp-up) before completing the final five trials in Block 2. This duration was to ensure a considerable level of stimulation was reached before performance began. The final 30 s of stimulation involved a “ramp-down” period. Participants were stimulated between a range of 15 and 21 min in total. This variation in stimulation time was due to the difference in time participants required to work through the familiarization stage of each trial in Block 2. Nevertheless, the stimulation duration and level of tDCS used in the present study remained well within safe limits (Bikson et al., 2009).

To ensure that participants were familiar with the experimental procedure, two complete practice trials were administered before the 10 experiment trials. The pieces of music in the practice trials were not used in the experiment trials. All performances were recorded using *GarageBand* (Version 10.2.0) on the MacBook Air and audio recordings were all formatted to ACC audio, de-identified, and randomly placed in a list of 160 performances for each judge to adjudicate.

### Expert Adjudication of Performances

The judges were provided with specific instructions and definitions for creativity and technical fluency to minimize ambiguity in judging. Creativity was defined as the quality of being *novel* and *appropriate* within a specific context. Technical fluency was defined as the level of accuracy and musicianship of the performances that may include accuracy in pitch and rhythm, articulation, and phrasing. Judges were instructed to rate the creativity and technical fluency of each of the 160 performances on two separate Likert scales ranging from 1 to 10. A score of 1 represented a low score on creativity or technical fluency; a score of 10 represented a very high level of creativity or technical fluency. Adjudicators were blind to the experimental conditions associated with each performance and did not know the true aim of the experiment or details about the participants’ musical background and training.

### Statistical Approach

To assess the consistency of ratings for creativity and technical fluency, a multiple-raters, consistency, 2-way mixed effects intra-class correlation coefficient (ICC) model was computed for the three independent judges across 16 participants. We conducted statistical tests to assess the reliability of differences in ratings of creativity and technical fluency between the two tDCS groups (excitatory vs. inhibitory). This comparison was first done for block one (no stimulation to either group) and again for block two (excitatory vs. inhibitory stimulation). To account for potential differences in the three judges’ assessments and differences as a function of the five consecutive attempts to improvise in each tDCS condition, we conducted two 2 × 3 × 5 mixed-ANOVAs, with Stimulation Group as the between-subjects factor (hereafter Group: excitatory or inhibitory), and Judge (1–3) and Take (1–5) as repeated measures factors. The first mixed-ANOVA analyzing the data from Block 1 was designed to check whether performances were similar across groups when no tDCS was administered. The second mixed-ANOVA analyzing the data from Block 2 was conducted to assess whether excitatory tDCS over the left M1 region resulted in performances that were rated by adjudicators as more creative and technically fluent than for those who received inhibitory tDCS. This approach ensured that all 960 data points from 16 participants were included in the analyses (160 performances rated by three adjudicators on creativity and technical fluency).

A Pearson’s *r* correlation coefficient was calculated to examine the association between mean creativity and technical fluency ratings averaged across the three judges. Structural analyses of improvisations were conducted and independent samples *t*-tests were computed to investigate any differences between the two tDCS groups with respect to the following three performance features: number of notes, pitch range, and number of different notes. Two multiple linear regressions were also computed to determine whether there was an association between these three performance features and ratings of creativity and technical fluency for the improvisations produced under conditions of tDCS in Block 2 (excitatory and inhibitory). Lastly, two components of sight-reading accuracy – pitch and timing accuracy – were recorded for all performances during the “sight-reading” stage of each trial. Timing accuracy was measured in milliseconds as an asynchrony between each performed note and the specific timing of each note as stipulated by the score. Pitch-note accuracy was coded as “0” each time participants pressed the correct piano key corresponding to each pitch in the score. Pitch-note accuracy was coded as “1” each time participants pressed the incorrect piano key relative to each note in the score. Therefore, the higher the score, the more inaccurate the sight-reading performance. Two independent samples *t*-tests were computed to analyze the sight-reading accuracy for both tDCS groups.

## Results

### Ratings of Creativity

The mean ICC for creativity was 0.507 with a 95% confidence interval from 0.358 to 0.626, *F*(159,318) = 2.029, *p* < 0.001. Therefore, inter-rater reliability for ratings of creativity across the three judges can be considered “fair” (Cicchetti, 1994). The first mixed-ANOVA analyzing the data from Block 1 was designed to assess whether ratings of performances were similar across groups when no tDCS was administered, as well as to monitor any differences between adjudicators or between the five consecutive improvisations in each condition. The second mixed-ANOVA analyzing the data from Block 2 assessed whether excitatory tDCS over the left M1 region resulted in performances that were rated by the adjudicators as more creative than for those who received inhibitory tDCS.

#### Block 1 (No Stimulation)

**FIGURE 2:**
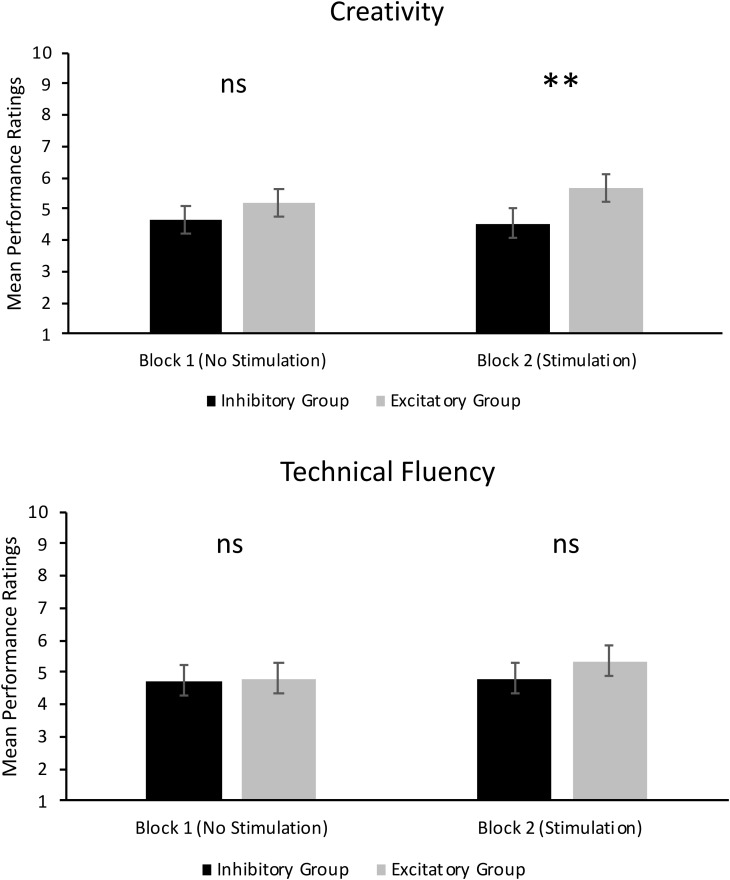
Mean performance ratings of creativity **(Top)** and technical fluency **(Bottom)**. Error bars report standard error of the mean. ^∗∗^*p* < 0.01.

As can be seen in the top panel of **Figure 2**, there was no significant main effect of Group in Block 1, *F*(1,14) = 1.21, *p* = 0.290, ${\text{η}}_{\text{p}}^{\text{2}}$ = 0.08. Thus, ratings of creativity in the excitatory tDCS group (*M* = 5.18, *SD* = 1.69) were not significantly different to ratings of creativity in the inhibitory tDCS group (*M* = 4.67, *SD* = 1.73) under conditions where no tDCS was administered. There was, however, a significant main effect of Judge in Block 1, *F*(2,28) = 20.97, *p* < 0.001, ${\text{η}}_{\text{p}}^{\text{2}}$ = 0.60. The mean rating of creativity from Judge 2 (*M* = 3.78, *SD* = 2.07) was significantly lower than that of Judge 1 (*M* = 5.43, *SD* = 1.63) and Judge 3 (*M* = 5.58, *SD* = 1.47, *p* < 0.001). There was no significant difference between mean ratings from Judge 1 and 3 (*p* = 0.511). There were no other significant effects.

#### Block 2 (Stimulation)

**FIGURE 3:**
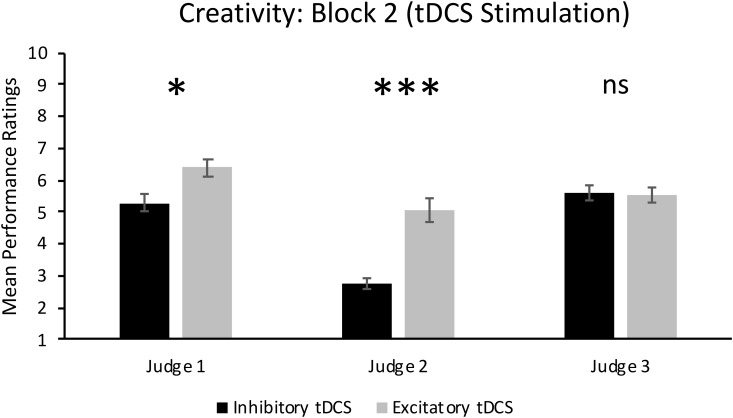
Individual judges’ ratings of creativity in Block 2 when participants received either excitatory or inhibitory tDCS. Error bars report standard error of the mean. ^∗^*p* < 0.05; ^∗∗∗^*p* = 0.001.

As can also be seen in the top panel of **Figure 2**, there was a significant main effect of Group in Block 2, *F*(1,14) = 10.50, *p* = 0.006, ${\text{η}}_{\text{p}}^{\text{2}}$ = 0.43. This result supports our hypothesis and shows that jazz improvisation performances by participants who received excitatory tDCS were rated significantly more creative (*M* = 5.68, *SD* = 1.80) than performances by participants who received inhibitory tDCS (*M* = 4.55, *SD* = 1.91). However, there was a significant Group × Judge interaction, *F*(2,28) = 10.35, *p* < 0.001, ${\text{η}}_{\text{p}}^{\text{2}}$ = 0.43. As can be seen in **Figure 3**, mean ratings of creativity were significantly greater in the excitatory tDCS condition relative to the inhibitory tDCS condition from Judge 1, *t*(14) = 2.35, *p* = 0.034, 95% CI [0.097, 2.153], and from Judge 2, *t*(14) = 4.46, *p* = 0.001, 95% CI [1.206, 3.444], but not from Judge 3, *t*(14) = -0.20, *p* = 0.844, 95% CI [-0.870, 0.720]. Overall, these findings appear to reflect both excitatory and inhibitory effects: six of the eight participants who received excitatory tDCS in Block 2 exhibited an absolute increase in rated creativity relative to Block 1 (no-stimulation), and four of the eight participants who received inhibitory tDCS in Block 2 exhibited an absolute decrease in rated creativity relative to Block 1.

### Ratings of Technical Fluency

The mean ICC for technical fluency was 0.475 with a 95% confidence interval from 0.317 to 0.602, *F*(159,318) = 1.906, *p* < 0.001. This result suggests that inter-rater reliability for ratings of technical fluency across the three judges can also be considered “fair” (Cicchetti, 1994). Similar to analyses of creativity, the first mixed-ANOVA analyzing the technical fluency data from Block 1 was designed to assess whether performances were similar across groups when no tDCS was administered, as well as to monitor any differences between adjudicators or between the five consecutive improvisations in each condition. The second mixed-ANOVA analyzing the data from Block 2 assessed whether excitatory tDCS over the left M1 region resulted in performances that were rated by the adjudicators as more technically fluent than for those who received inhibitory tDCS.

#### Block 1 (No Stimulation)

As can be seen in the bottom panel of **Figure 2**, there was no significant main effect of Group in Block 1, *F*(1,14) = 0.05, *p* = 0.832, ${\text{η}}_{\text{p}}^{\text{2}}$ = 0.00. Thus, ratings of technical fluency were not significantly different between the excitatory tDCS group (*M* = 4.82, *SD* = 1.81) and the inhibitory tDCS group (*M* = 4.74, *SD* = 1.76) under conditions where no tDCS was administered. There was also a significant main effect of Judge, *F*(2,28) = 44.22, *p* < 0.001, ${\text{η}}_{\text{p}}^{\text{2}}$ = 0.76. Similar to ratings of creativity, the mean rating of technical fluency from Judge 2 (*M* = 3.28, *SD* = 1.94) was significantly lower than Judge 1 (*M* = 5.69, *SD* = 1.58) and Judge 3 (*M* = 5.34, *SD* = 1.32, *p* < 0.001). There was no significant difference between Judge 1 and Judge 3 (*p* = 0.100). There were no other significant effects.

#### Block 2 (Stimulation)

As can be seen in the bottom panel of **Figure 2**, there was no significant main effect of Group in Block 2, *F*(1,14) = 2.28, *p* = 0.153, ${\text{η}}_{\text{p}}^{\text{2}}$ = 0.14. Thus, the type of tDCS administered to participants did not differentially affect the technical fluency of their performances. However, there was a significant main effect of Judge, *F*(2,28) = 38.59, *p* < 0.001, ${\text{η}}_{\text{p}}^{\text{2}}$ = 0.73, which followed the same trend in results as the aforementioned main effects of Judge, and a significant Judge × Take interaction, *F*(8,112) = 2.39, *p* = 0.021, ${\text{η}}_{\text{p}}^{\text{2}}$ = 0.15. Examination of mean ratings suggests that this interaction may be driven by differences in ratings between judges at take 5. Judges 1 and 3 assigned similar overall ratings of technical fluency (and creativity) across takes 1–4. However, mean ratings of technical fluency by Judge 3 dropped below that of Judge 1 in take 5.

### Correlation Between Ratings of Creativity and Technical Fluency

All trials were analyzed irrespective of tDCS stimulation to investigate the relationship between creativity and technical fluency. For Judge 1, there was a significant positive correlation between technical fluency and creativity, *r* = 0.72, 95% BCa CI [0.621, 0.794], *p* = 0.01. For Judge 2, there was also a significant positive correlation between technical fluency and creativity, *r* = 0.74, 95% BCa CI [0.633, 0.850], *p* = 0.01. Finally, for Judge 3 there was a significant positive correlation between technical fluency and creativity, *r* = 0.67, 95% BCa CI [0.578, 0.741], *p* = 0.01. All reported correlations are considered to reflect a large effect size (Babchishin and Helmus, 2016).

### Melodic Performance Features

#### Total Number of Notes Used

The analysis revealed a significant difference between the excitatory tDCS group (*M* = 30.28, *SD* = 5.26) and the inhibitory tDCS group (*M* = 21.23, *SD* = 4.31), *t*(14) = 3.763, *p* = 0.002. This result shows that with tDCS stimulation to the M1, the mean total number of notes used in the improvisation stage was significantly greater for those who experienced excitatory tDCS when compared to inhibitory tDCS.

#### Number of Different Notes Used

The analysis revealed a significant difference between the excitatory tDCS group (*M* = 9.00, *SD* = 0.76) and the inhibitory group (*M* = 7.83, *SD* = 1.05), *t*(14) = 2.569, *p* = 0.022. This result shows that when tDCS stimulation is applied to the M1, the mean number of different notes used in the improvisation stage was significantly greater for those who experienced excitatory tDCS when compared to inhibitory tDCS.

#### Pitch Range

The analysis also revealed a significant difference between the excitatory tDCS group (*M* = 19.93, *SD* = 5.53) and the inhibitory group (*M* = 14.20, *SD* = 1.54), *t*(8) = 2.288, *p* = 0.022. This result shows that when tDCS stimulation is applied to the M1, the mean pitch range used in the improvisation stage was significantly larger for those who experienced excitatory tDCS when compared to inhibitory tDCS.

### Association Between Creativity, Technical Fluency, and Melodic Performance Features

#### Ratings of Creativity

For the *excitatory* tDCS group in Block 2, the three melodic performance features (total number of notes, number of different notes, and pitch range) were significant predictors of creativity, *F*(3,4) = 8.381, *p* = 0.034, Adjusted *R*^2^= 0.760. For the *inhibitory* tDCS group in Block 2, the three melodic performance features were not significant predictors of creativity, *F*(3,4) = 2.632, *p* = 0.186, adjusted *R*^2^= 0.412.

#### Ratings of Technical Fluency

For the *excitatory tDCS* group in Block 2, the three melodic performance features were not significant predictors of technical fluency *F*(3,4) = 3.149, *p* = 0.148, adjusted *R*^2^ of 0.479. For the *inhibitory* tDCS group in Block 2, the three melodic performance features were also not significant predictors of technical fluency, *F*(3,4) = 0.1479, *p* = 0.906, adjusted *R*^2^ of -0.543.

### Sight-Reading Performance Accuracy

#### Timing Accuracy

In the sight-reading stage of performances in Block 1 (section A in each score), the analysis revealed no significant difference in timing accuracy between the excitatory tDCS group (*M* = 27.81 ms, *SD* = 49.22) and the inhibitory tDCS group (*M* = 87.74 ms, *SD* = 140.95), *t*(14) = -1.135, *p* = 0.287. In the sight-reading stage of performances in Block 2, the analysis also revealed no significant difference between the excitatory tDCS group (*M* = 12.70 ms, *SD* = 49.43) and the inhibitory tDCS group (*M* = 93.15 ms, *SD* = 139.93), *t*(14) = -1.533, *p* = 0.161. These results show that tDCS stimulation did not significantly affect timing accuracy in the sight-reading stage.

#### Pitch-Note Accuracy

In the sight-reading stage of performances in Block 1, the analysis revealed no significant difference in pitch-note accuracy between the excitatory tDCS group (*M* = 0.55, *SD* = 0.72) and the inhibitory tDCS group (*M* = 1.24, *SD* = 1.68), *t*(14) = -1.062, *p* = 0.314. In the sight-reading stage of performances in Block 2, the analysis also revealed no significant difference between the excitatory tDCS group (*M* = 0.42, *SD* = 0.36) and the inhibitory tDCS group (*M* = 1.18, *SD* = 2.02), *t*(14) = -0.950, *p* = 0.385. This result shows that the type of tDCS stimulation did not affect pitch-note accuracy in the sight-reading section.

## Discussion

The aim of this investigation was to determine whether the M1 plays a role in creative and technically fluent musical improvisations. Expert jazz pianists received either excitatory or inhibitory tDCS over the left hemispheric M1 while completing right-handed jazz piano performances that comprised a sight-reading stage and an improvisation stage. Performances were adjudicated by expert musicians who judged creativity and technical fluency. We hypothesized that improvisations performed by participants who received excitatory tDCS would be more creative and technically fluent than improvisations performed by those who received inhibitory tDCS. This hypothesis was supported for ratings of creativity: improvisations by participants who received excitatory tDCS were rated as significantly more creative than those who received inhibitory tDCS. Interestingly, we observed no significant differences between excitatory and inhibitory tDCS for ratings of technical fluency. Follow-up analyses revealed that melodic performance features such as the total number of notes played, number of different notes played, and pitch range were significant predictors of creative performances for those in the excitatory tDCS group. The type of tDCS did not differentially affect sight-reading accuracy as measured by timing and pitch-note accuracy in the sight-reading stage.

One possible explanation for the results is that the M1 mediates the potential for a creative motor action associated with a pre-planned creative idea. Specifically, the foundations of a creative idea may form in brain areas associated with higher-order creative processes such as attention, planning, working memory, cognitive flexibility, and imagination, and then flow in part via the M1 to be realized as a creative motor action (Dietrich, 2004; Lucchiari et al., 2018). Research suggests that networks in the PFC are responsible for higher-order cognitive functions (specifically the ECN and DMN) associated with creative processes in all domains including music (Bengtsson et al., 2007; Gonen-Yaacovi et al., 2013; Boccia et al., 2015). It is also clear that the PFC and the M1 are functionally linked (e.g., Hasan et al., 2013). Thus, exciting the M1 may have increased the potential for converting a preplanned creative idea into a creative motor action. In the context of piano improvisations in the present study, stimulating the M1 may have facilitated the flow of creative “content” (notes) from the pre-planned creative idea into motor output (piano performance). As a result, improvisations during excitatory tDCS were more creative than inhibitory tDCS because they reflected an increased output of creative performance features.

The data reported here provide some support for this interpretation. Participants who received excitatory tDCS performed improvisations with a significantly greater number of notes and greater number of different notes, as well as a wider pitch range than participants who received inhibitory tDCS. Furthermore, results from multiple regression analyses showed that these three performance features were significant predictors of creativity for the excitatory tDCS group, explaining 76% of the variance. This was not the case for the inhibitory tDCS group.

Interestingly, a parallel effect of tDCS on technical fluency was not observed, even though ratings of creativity and technical fluency were positively and significantly correlated. Brain stimulation may have facilitated the flow of creative ideas from higher levels of processing through to motor planning and motor actions, releasing a low-level neural “gateway” for high level creative ideas. Technical fluency, in contrast, may operate independently of that process of disinhibition and may instead rely on over-learned, automated processes of action control that are comparatively fixed through training and less susceptible to transient changes from stimulation. Alternatively, it may be that task demands for technical fluency were such that there was less opportunity for performers to differ in technical fluency than in creativity. For performers to display fluency, they needed to play syntactically plausible pitches on plausible metric subdivisions. Although timing and pitch errors occurred, the task demands might have afforded less opportunity for variability in technical fluency.

Finally, judges evaluated the inherently creative musical task of improvisation. As a result, they may have focused more attention and greater cognitive resources on their judgments of creativity and fewer resources on the adjudication of technical fluency, thus resulting in less reliability in judgments of fluency. Future research could alleviate this possibility by recruiting two groups of judges: one that adjudicates the creative element of each performance, and the other that adjudicates technical fluency. Indeed, the difference in results between creativity and technical fluency will need to be replicated in future studies with greater statistical power by including more expert performers and adjudicators. Nevertheless, there was a strong positive correlation between creativity and technical fluency ratings from all three adjudicators irrespective of the type of tDCS stimulation participants received. This result suggests that creativity and technical fluency are related phenomena in adjudication of musical improvisation, even though both are differentially affected by stimulation of the M1.

To investigate the M1 with greater localization specificity, future studies could also use a rTMS paradigm. rTMS is a non-invasive brain stimulation technique that facilitates or inhibits neural activity by modulating MEPs in the M1 (Romero et al., 2002; Peinemann et al., 2004). This is accomplished by varying the frequency of pulses (pulses per second), number of total pulses, and the inter-train interval (period where TMS is not administered). rTMS has the potential to modulate neural activity for a prolonged period (20–60 min) and with greater localization specificity than tDCS (Huang et al., 2005; Rotenberg et al., 2014). Replicating the present study with rTMS will allow more causal inferences to be made regarding the role of the M1 region in creative and technically fluent piano improvisations.

## Conclusion

To conclude, our findings illustrate an important role for the M1 in musical creativity. Indeed, the M1 may not only act as a gateway for translating creative cognition into action, but likely mediates the potential for maximizing such creative output. Although more research is needed to link such an association to applied contexts such as performance pedagogy, the results imply that programs emphasizing movement and rhythm have the potential to benefit creative musicianship. Technical fluency, on the other hand, may operate independently of this process and instead rely on learned automated actions that are comparatively fixed through music training. Future research is needed to evaluate these proposals with a greater number of expert musician participants and adjudicators. Nevertheless, the current findings suggest that the M1 should receive greater consideration in the already complex neural network that mediates creativity, especially in the context of movement-based expertise.

## Author Contributions

AA coordinated testing and data collection. AA and KO were responsible for data analysis and all authors contributed to data interpretation. AA wrote the first draft of the manuscript and all authors contributed to further revisions. All authors approved the final version of the manuscript, and contributed to the design and development of the study.

## Conflict of Interest Statement

The authors declare that the research was conducted in the absence of any commercial or financial relationships that could be construed as a potential conflict of interest.

## Abbreviations

ACC: anterior cingulate cortex
AIC: anterior insula cortex
DLPFC: dorsolateral prefrontal cortex
DMN: default mode network
dPMC: dorsal premotor cortex
ECN: executive control network
EEG: electroencephalography
fMRI: functional magnetic resonance imaging
IHIC: inter-hemispheric inhibition connection
M1: primary motor cortex
MEP: motor-evoked potential
PCC: posterior cingulate cortex
PFC: prefrontal cortex
pre-SMA: pre-supplementary motor area
rTMS: repetitive transcranial magnetic stimulation
TMS: transcranial magnetic stimulation
tDCS: transcranial direct current stimulation
vMPFC: ventromedial prefrontal cortex
vPMC: ventral premotor cortex
