# Supplementary figures and images for: Investigating the Role of the Primary Motor Cortex in Musical Creativity: A Transcranial Direct Current Stimulation Study
Source: Front Psychol. 2018 Oct 1;9:1758. doi: 10.3389/fpsyg.2018.01758 (PMC6174363; doi:10.3389/fpsyg.2018.01758)

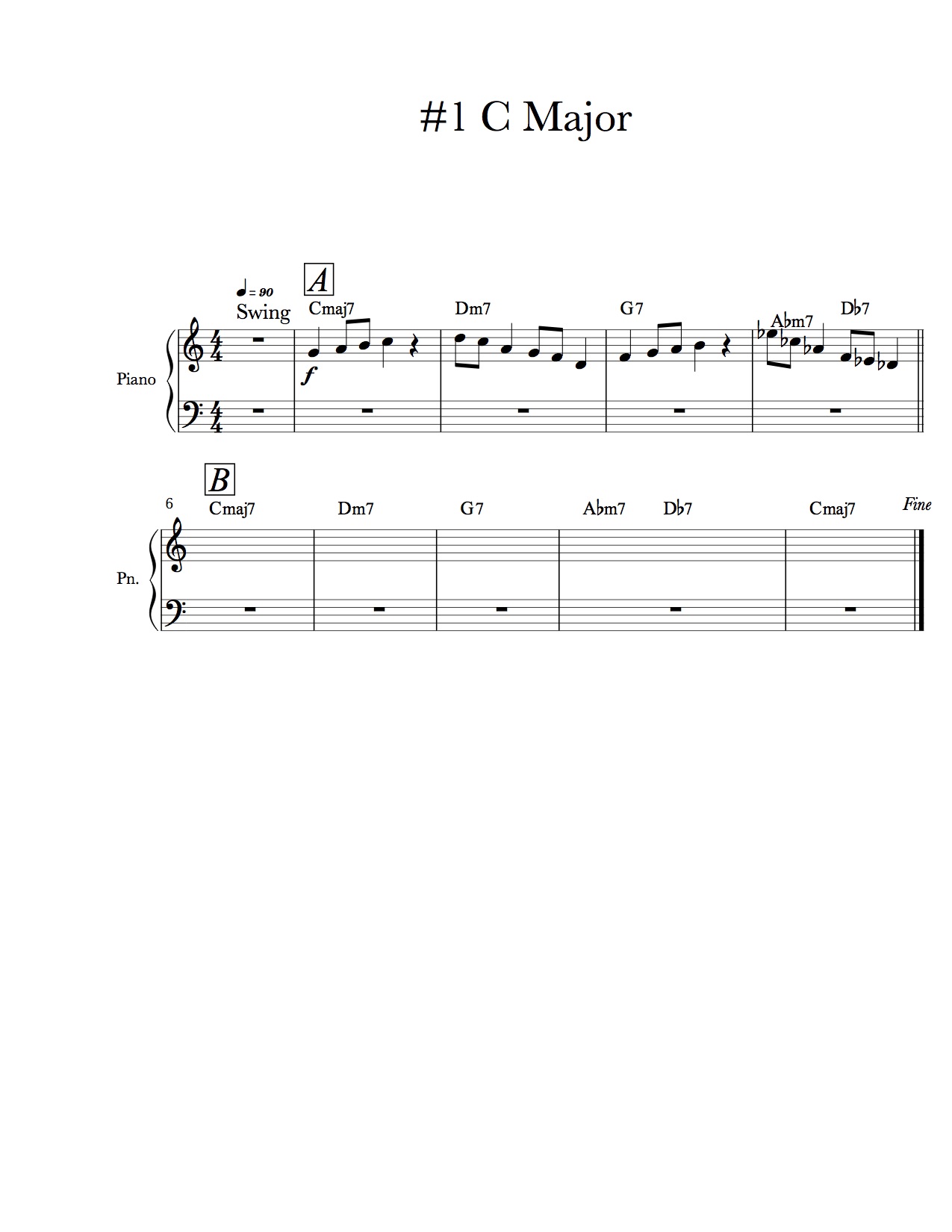

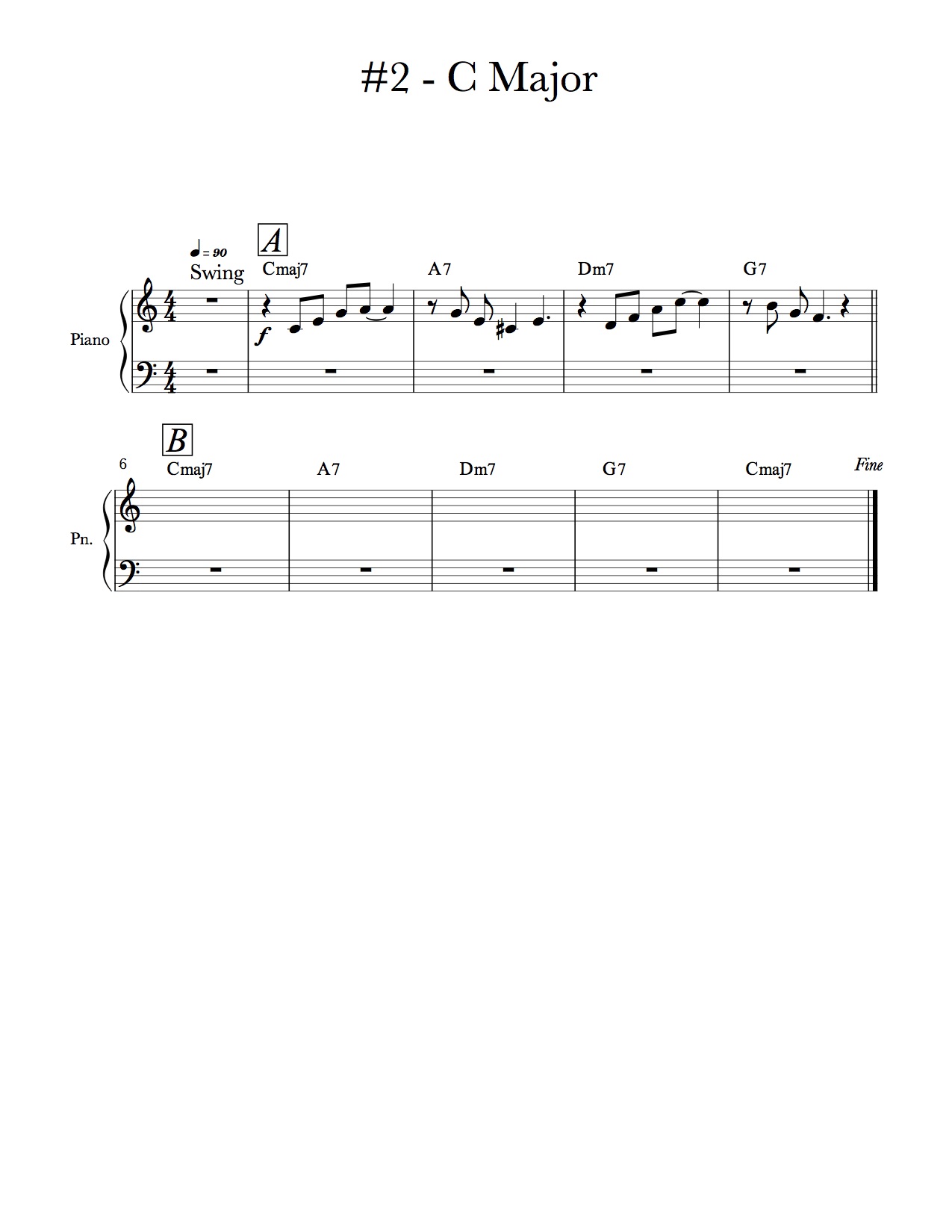

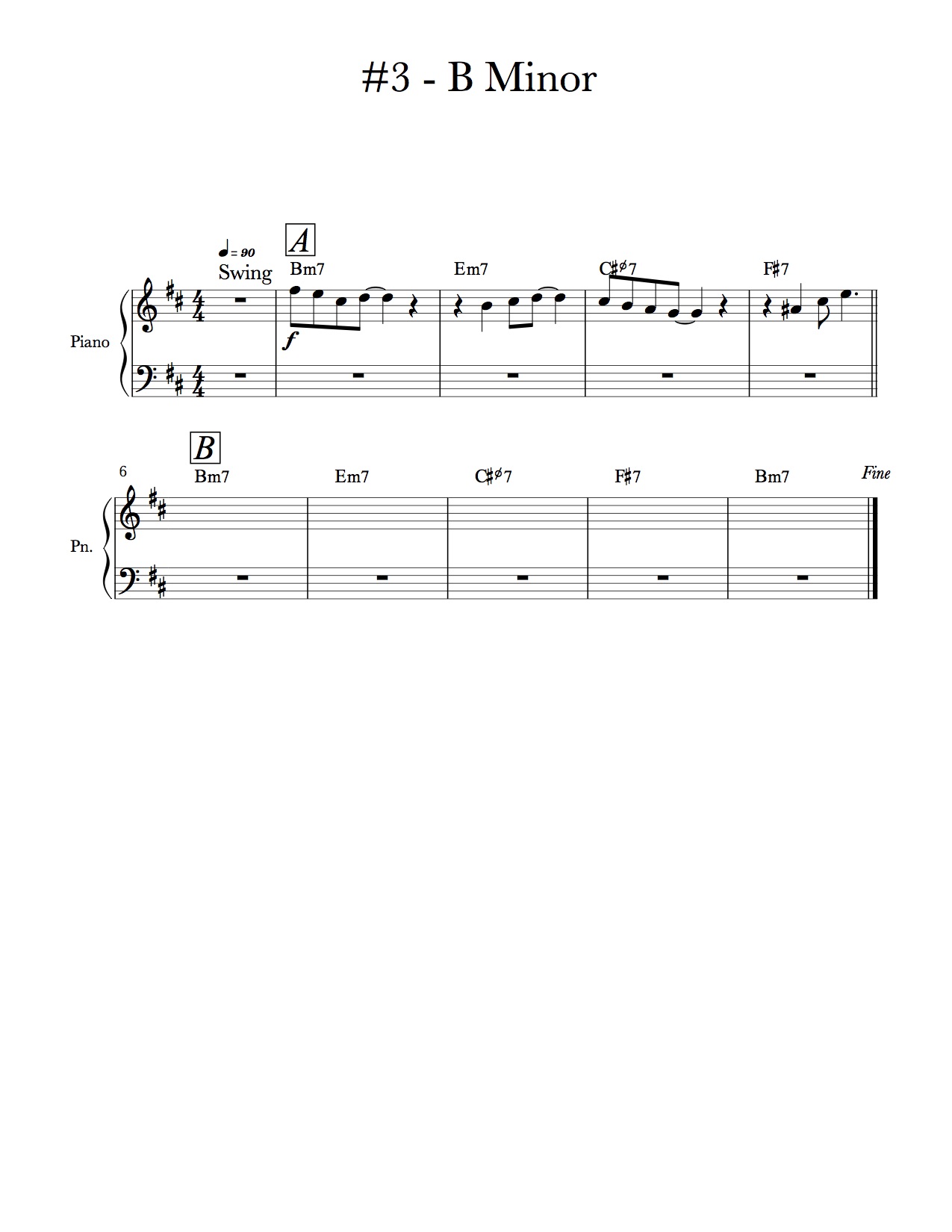

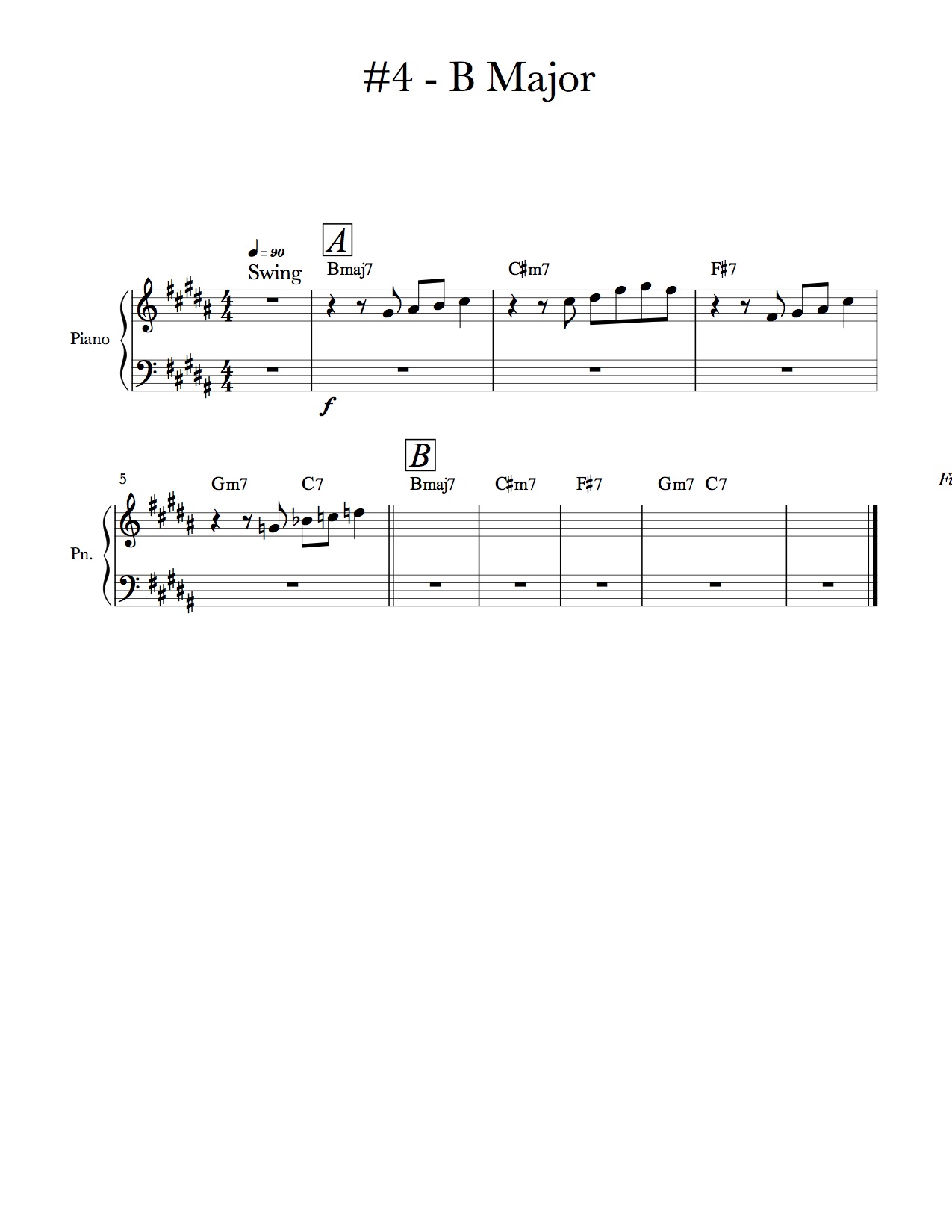

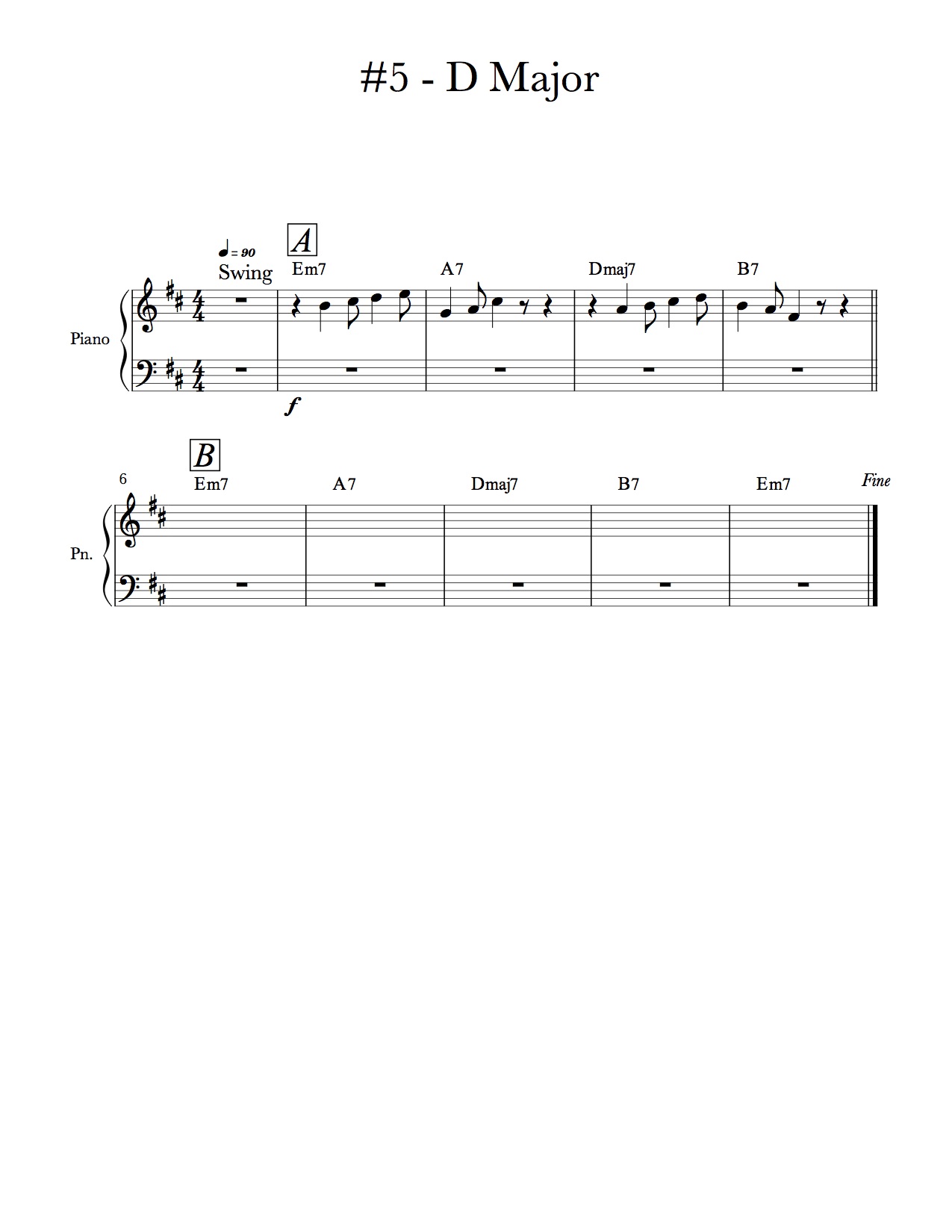

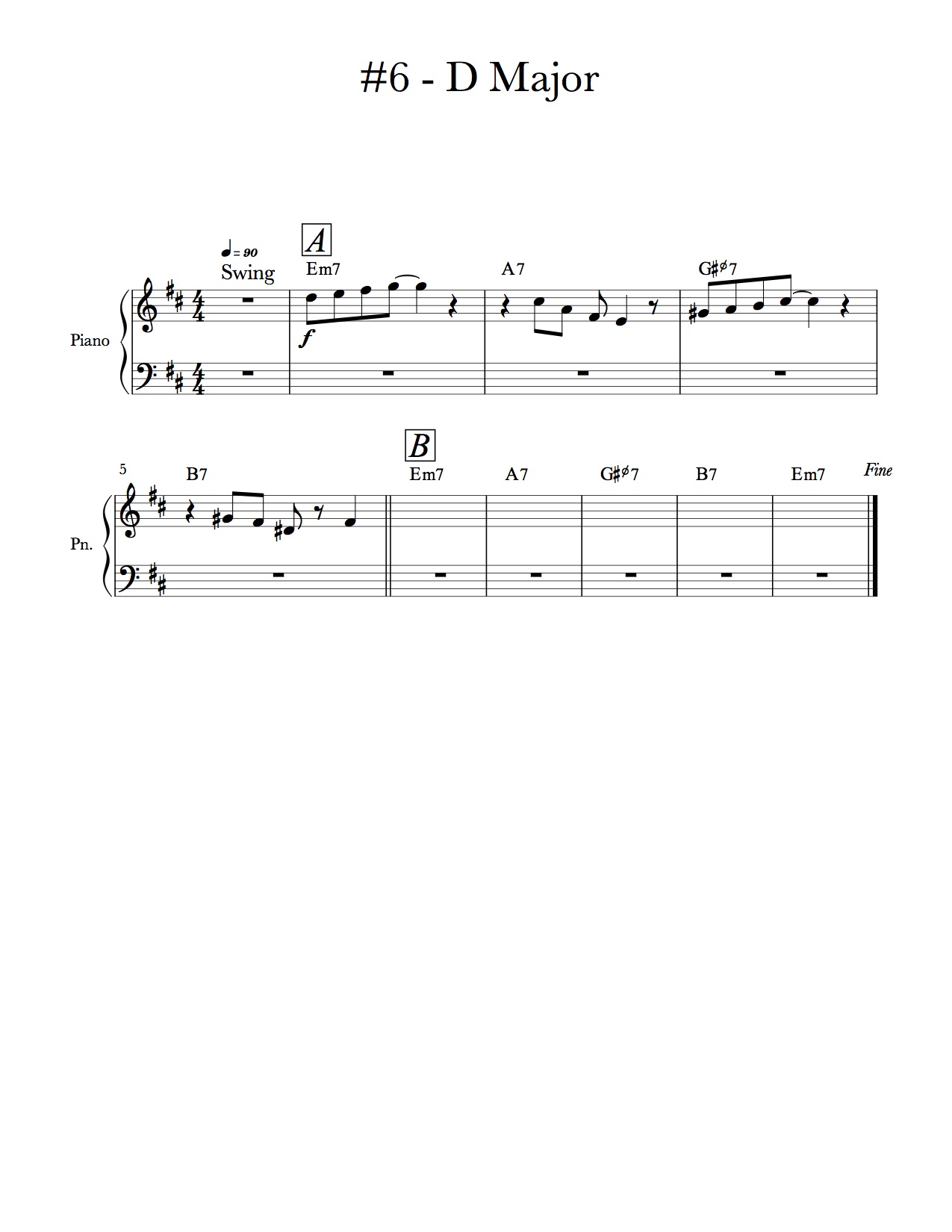

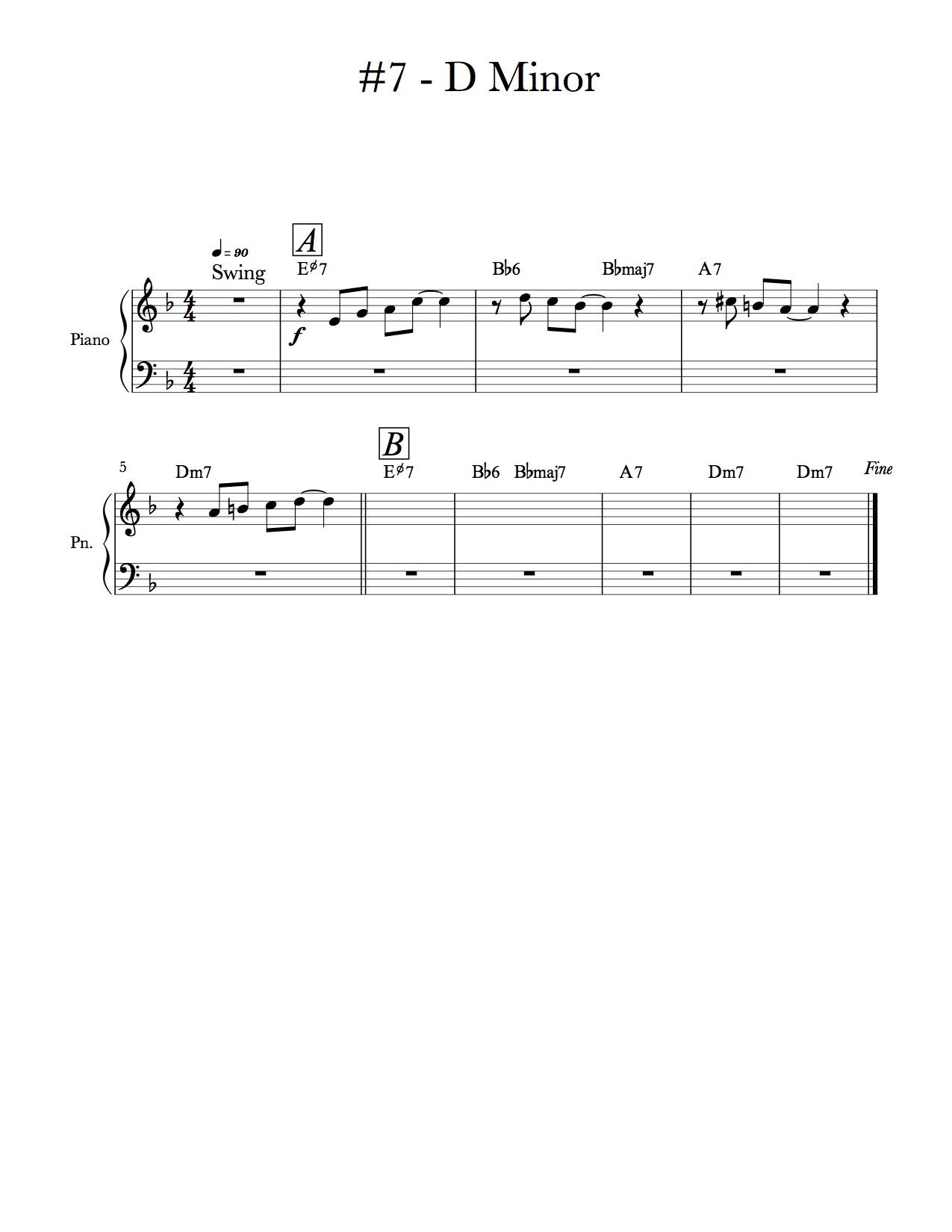

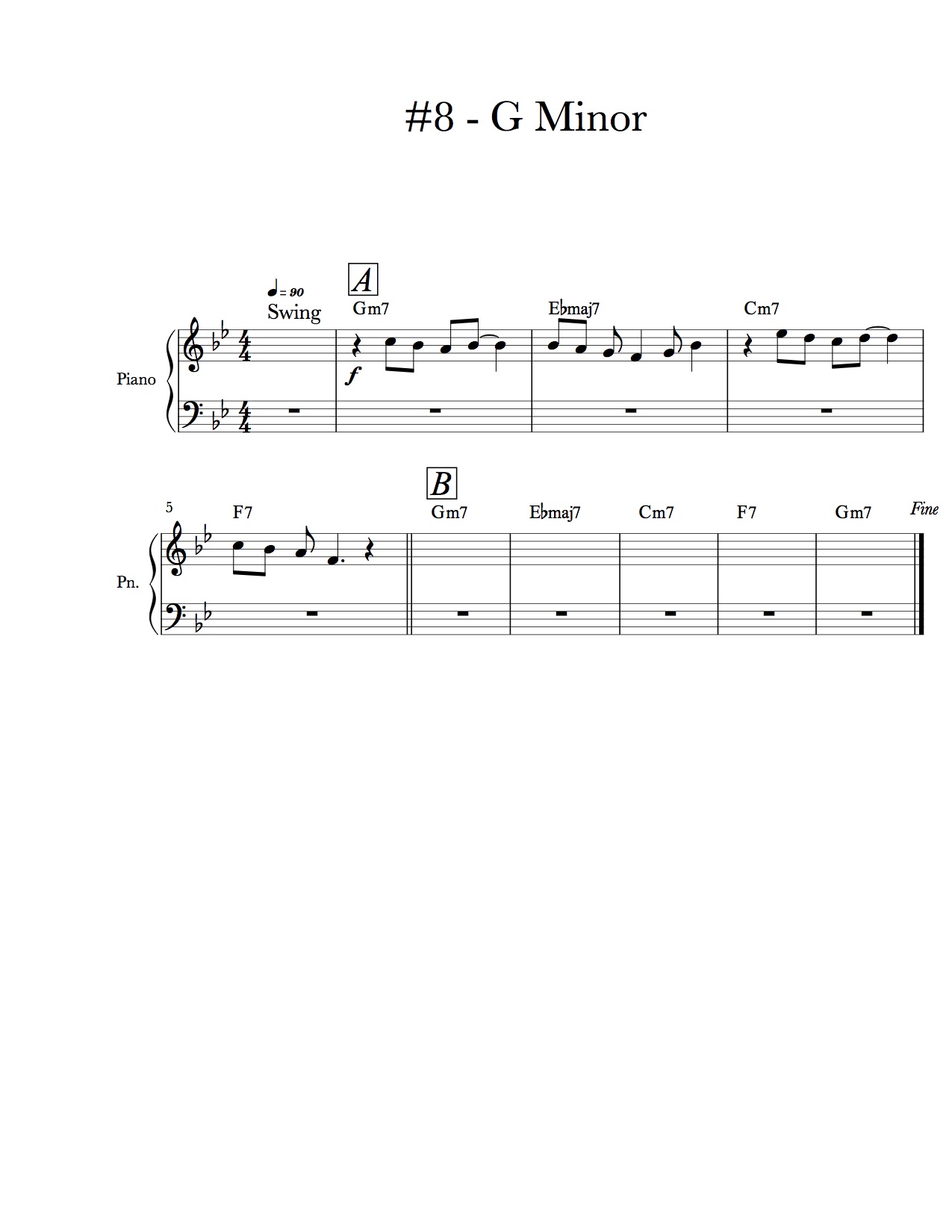

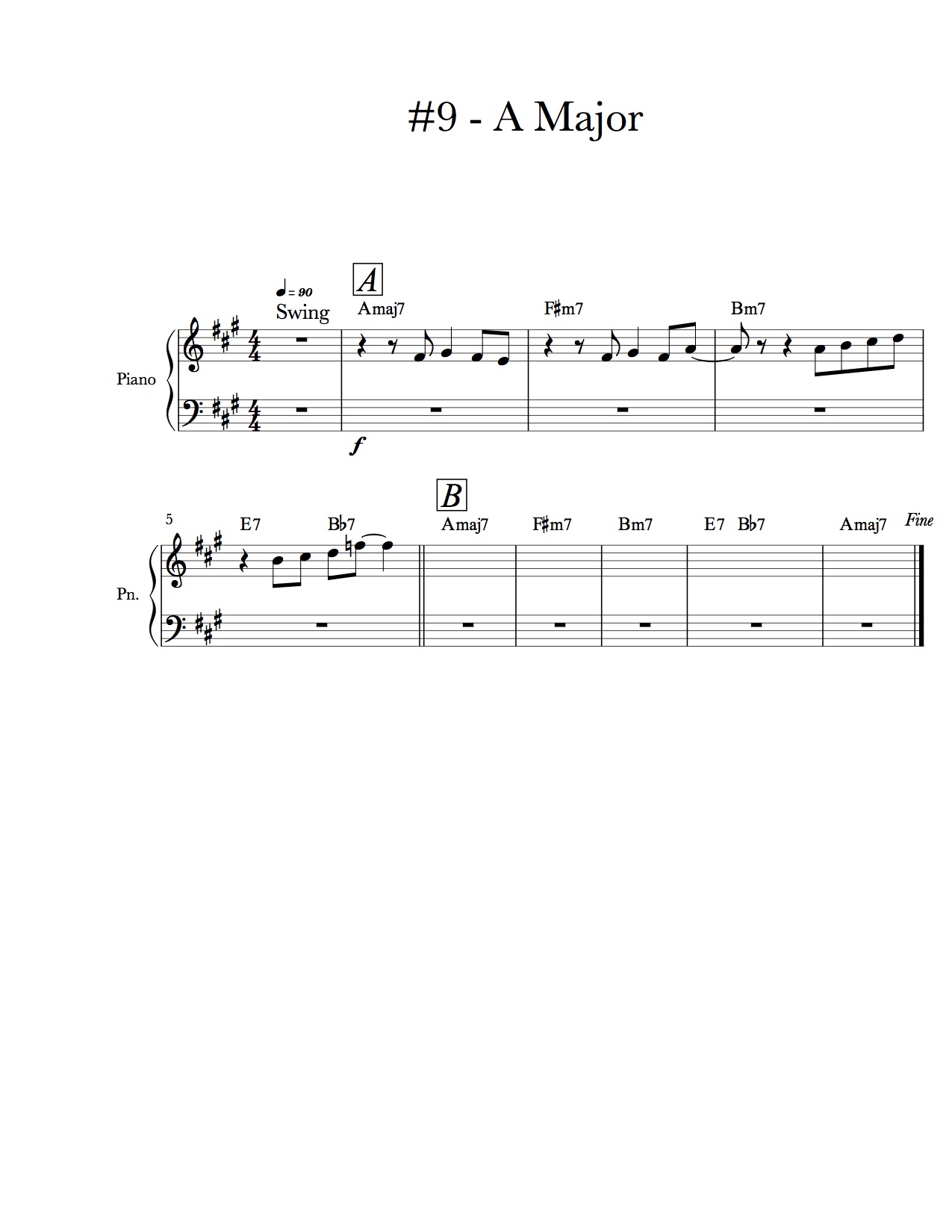

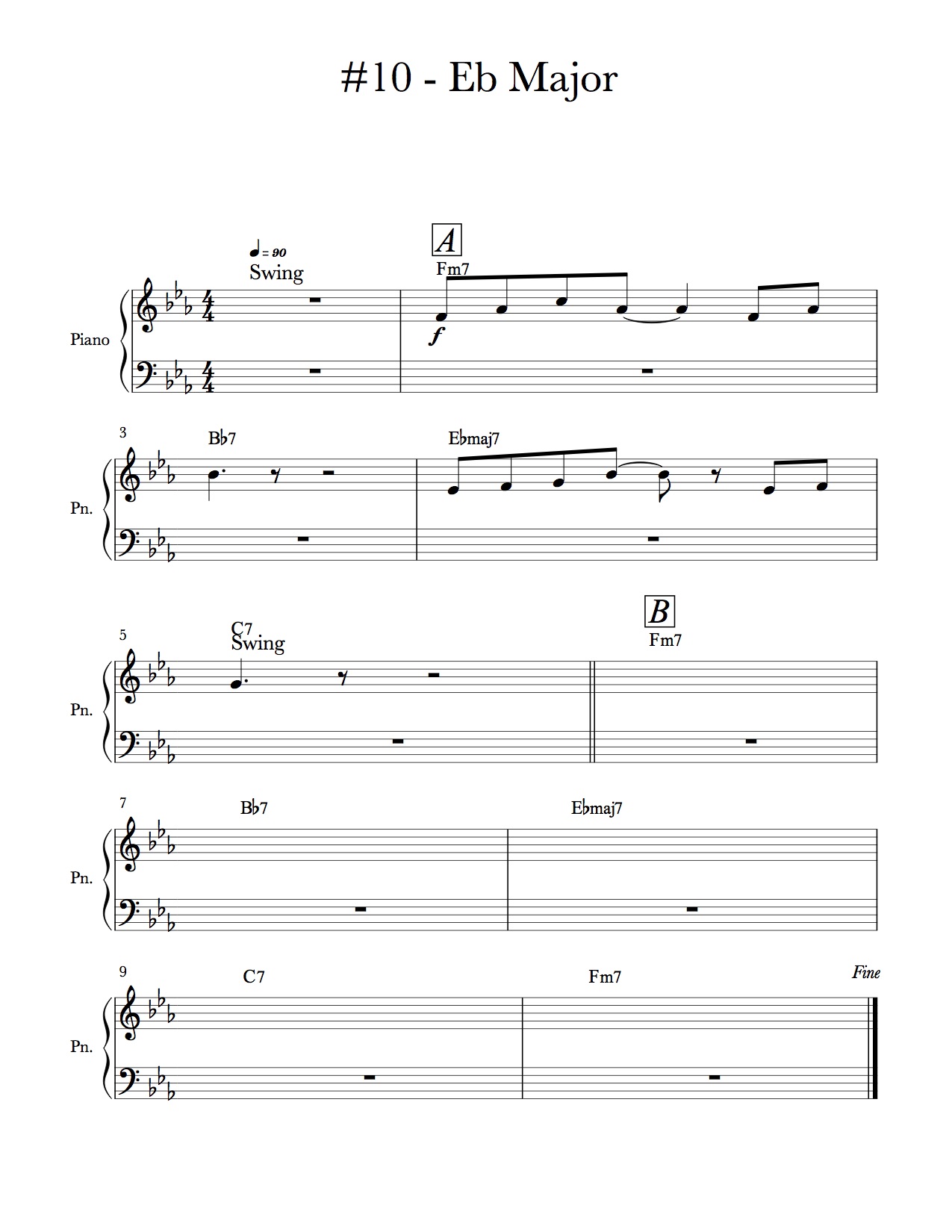

Supplement: Supplementary file 1 [file Table_1.DOCX]
